# Are There Unique Barriers and Opportunities for Access to Endoscopic Spine Surgery in Low-Income Countries? A Narrative Review

**DOI:** 10.3390/jcm14113876

**Published:** 2025-05-30

**Authors:** Adham M. Khalafallah, Sara Diez, Long Di, Saqib Hasan, Sanjay Konakondla, Osama N. Kashlan, Peter Derman, Mark Mahan, Raymond J. Gardocki, Albert Telfeian, Christoph P. Hofstetter, Gregory Basil

**Affiliations:** 1 Department of Neurosurgery, University of Miami, Miami, FL 33136, USA; long.di@jhsmiami.org (L.D.); gbasil@med.miami.edu (G.B.); 2 College of Medicine, Florida State University, Tallahassee, FL 32306, USA; saradieza@gmail.com; 3 Golden State Orthopedics and Spine, Oakland, CA 94609, USA; saqibhasanmd@gmail.com; 4 Department of Neurosurgery, Geisinger Neuroscience Institute, Danville, PA 17822, USA; skonakondla2@geisinger.edu; 5 Department of Neurological Surgery, Och Spine at NewYork-Presbyterian Hospital, Weill Cornell Medicine, New York, NY 10034, USA; onk4001@med.cornell.edu; 6 Department of Spine Surgery, Texas Back Institute, Plano, TX 75093, USA; peter.derman@gmail.com; 7 Department of Neurosurgery, Clinical Neurosciences Center, University of Utah, Salt Lake City, UT 84132, USA; mark.mahan@hsc.utah.edu; 8 Department of Orthopaedic Surgery, Vanderbilt University Medical Center, Nashville, TN 37232, USA; 9 Department of Neurosurgery, Warren Alpert Medical School, Brown University, Providence, RI 02903, USA; atelfeian@brownhealth.org; 10 Department of Neurological Surgery, University of Washington, Seattle, WA 98104, USA; chh9045@uw.edu

**Keywords:** endoscopic spine surgery, low-income countries, neurosurgical access, global health equity

## Abstract

Full endoscopic spine surgery (FESS) offers an ultra-minimally invasive solution for addressing many different degenerative spine pathologies. While FESS has demonstrated strong evidence for faster recovery, reduced hospital stays, fewer complications, and potentially lower overall costs, FESS remains underutilized in low-income countries (LICs). This narrative review synthesizes the existing literature to evaluate access to FESS in LICs, highlighting challenges such as a lack of trained neurosurgeons and orthopedic surgeons, insufficient access to specialized equipment, capital costs, and limited representation in research. A systematic literature search identified only a handful of relevant studies, underscoring the scarcity of data on FESS in LICs. Findings reveal stark disparities in training opportunities and equipment availability, with less than 25% of LIC facilities equipped with the essential tools. This review advocates for international collaboration, increased funding, cost reduction, and targeted research to bridge these gaps. Innovative solutions such as virtual training platforms may help overcome current limitations. Addressing these challenges is essential to leveraging FESS’s potential to mitigate the burden of spinal disorders in LICs and advance global health equity.

## 1. Introduction

Globally, disorders of the spine are a leading cause of disability and reduced quality of life, posing a significant burden on healthcare systems and global productivity [1]. While substantial progress has been made in socio-economic development over the past two decades, low-income countries (LICs), as defined by the World Bank Index, remain disproportionately affected by healthcare inequalities. The number of countries classified as LICs has decreased from 66 in 2003 to 21 in 2023, yet the populations in these nations continue to face substantial challenges due to limited resources and underrepresentation in research and development [2].

LICs experience a heightened burden of degenerative spinal pathologies, compounded by fewer resources compared to high-income countries. As of 2018, it was estimated that low- and middle-income countries had four times more cases of degenerative spine pathologies than high-income countries [3]. Many of these conditions, including disk herniation, spondylolisthesis, and lumbar spinal stenosis, can often be effectively treated with endoscopic approaches to the spine.

FESS is gaining traction in high-income countries due to its ability to minimize hospital stays, expedite recovery, and reduce complication rates compared to traditional open surgery [4]. Many of the benefits of spinal endoscopy can be attributed to the minimal disruption of normal anatomy, resulting in lower pain, quicker patient recovery, and often same-day discharges [5,6,7]. Additionally, FESS’s lower complication rates and reduced need for follow-up visits contribute to decreased overall healthcare costs [8].

Despite these advantages, the adoption of FESS in LICs remains limited. Barriers such as a longer learning curve, limited training opportunities, and a lack of costly specialized equipment hinder its implementation. Ironically, these settings could benefit the most from FESS’s cost-efficiency and recovery benefits. This narrative review examines the current state of access to FESS in LICs, analyzes the literature available on this topic, and identifies the primary barriers to implementing FESS in these underserved regions.

## 2. Materials and Methods

A systematic narrative review was conducted in accordance with PRISMA guidelines to evaluate access to FESS in LICs. A PubMed search was performed to identify relevant studies published within the past 10 years. Articles were included if they addressed FESS, defined as a minimally invasive technique using a uniportal endoscope to treat spinal pathologies, and LICs, classified per the World Bank’s 2024 income classification [2].

The search strategy utilized keywords including “endoscopic spine surgery” and “low-income countries”. MeSH terms were unavailable for FESS; however, related terms such as “neurosurgery” and “neuroendoscopy” were explored. A query combining “neuroendoscopy” and “low-income countries” yielded 20 articles, none of which directly addressed FESS, leading to their exclusion. A separate search using “endoscopic spine surgery” and “low-income countries” resulted in six articles, of which four were deemed relevant after screening. One article was excluded for focusing on exoscopic rather than endoscopic techniques. One additional article was identified through a manual reference review. While this study follows PRISMA guidelines for transparency in reporting, the heterogeneity and limited number of studies warranted a narrative synthesis rather than a formal systematic review.

**Figure 1:**
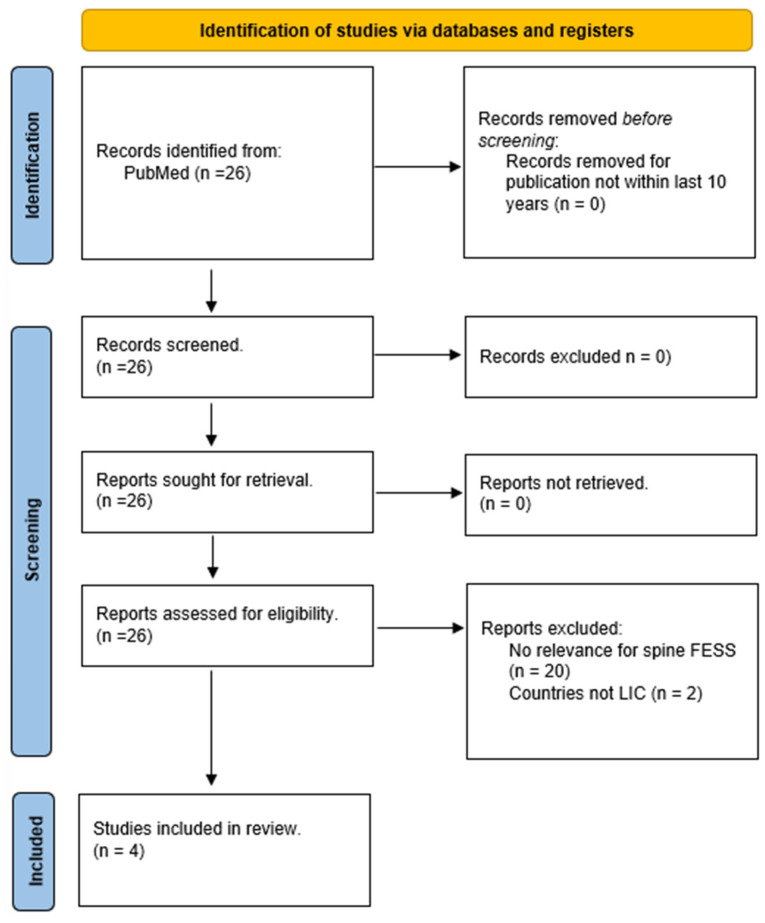
Systematic review flow diagram using Preferred Reporting Items for Systematic Reviews guidelines.

An article addressing the global neurosurgical workforce was included as a proxy for access to specialized surgical techniques in LICs. Supplementary data from World Bank publications were incorporated to contextualize findings. The literature search and article selection process are summarized in Figure 1.

## 3. Results

A comprehensive review of the literature revealed profound disparities in access to FESS in LICs, particularly concerning workforce distribution, equipment availability, and research representation. From an initial keyword search, six articles were identified, with four meeting the inclusion criteria. An additional study identified through manual reference review brought the total to five. These studies provided data from LICs in two geographic regions—Sub-Saharan Africa and Southeast Asia—offering insights into the availability of neurosurgeons and access to FESS (Table 1).

### 3.1. Neurosurgical Workforce Distribution

**Figure 2:**
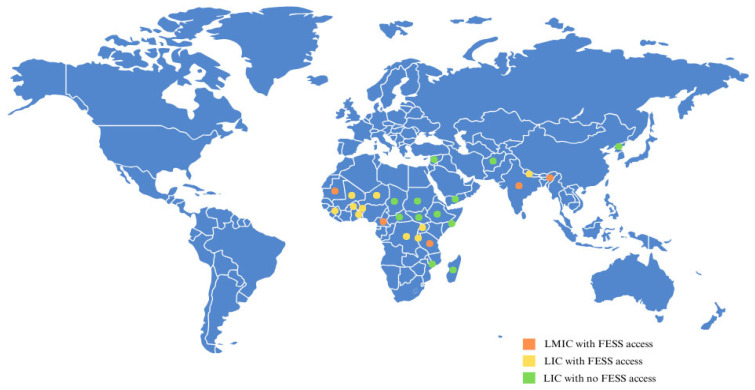
Map the distribution of access to FESS in low- and lower-middle-income countries (LICs and LMICs).

The included studies spanned 198 countries, with a specific emphasis on 12 LICs in Sub-Saharan Africa and additional LIC regions in Southeast Asia (Figure 2). Rahman et al. estimated a global neurosurgical workforce of approximately 49,940 neurosurgeons but highlighted severe regional disparities [9]. The lowest densities of neurosurgeons were observed in Sub-Saharan Africa (0.51 neurosurgeons per million people) and Southeast Asia (2.59 per million), compared to significantly higher densities in high-income regions such as the United States (14.7 per million). Notably, 33 countries—including five LICs—had no neurosurgeons at all. Gupta et al. reinforced these disparities, showing that none of the LICs met the global target of one neurosurgeon per 100,000 people, in contrast to 50.8% of high-income countries (HICs) achieving this benchmark [10]. These findings underscore severe limitations in the availability not only of basic Neurosurgical care but also of specialized procedures such as FESS in LICs, where the shortage of trained professionals remains a critical barrier.

**Table 1:** Summary of articles meeting inclusion criteria. High-income country (HIC), upper middle-income country (UMIC), lower middle-income country (LMIC), low-income country (LIC), Sub-Saharan Africa (SSA).

Article	Total Countries	LIC	Year of Publication	Number	Results of Study
Gupta et al. [10]	192	23 divided into regions in SSA and SE Asia	2024	177 survey participants	This study looked at the neurosurgeon workforce distribution globally. They found the global density to be 0.93 neurosurgeons per 100,000 people. The study described that the workforce is expanding toward the target of one neurosurgeon per 100,000 people. The study found that the target distribution was met by 50.8%, 32.1%, 7.7%, and 0% of HICs, UMICs, LMICs, and LICs, respectively. The disparities in density by national income level are therefore still vast [10].
Karekezi et al. [11]	18	12 all in SSA	2020	21 surveyed participants	Data collected from World Federation Neurosurgical Societies Rabat Training Center recent graduates looked at the resource availability in LICs in Sub-Saharan Africa. This paper found that equipment availability varied greatly: a CT scanner was available to 86%, MRI to 38%, surgical microscope to 33%, endoscope to 19.1%, and neuronavigation to 0%. Three neurosurgeons (14.3%) had access to none of the above [11].
Liu et al. [12]	43	Not explicitly mentioned	2023	990 articles	A bibliometric study found growing trends in research on full-endoscopic spine decompression with an increasing number of international collaborations; the countries found to have the most publications were of high- and middle-income status [12].
Wu et al. [13]	53	Not explicitly mentioned	2022	1196 articles	This study looked at the most productive nations in publications on endoscopic discectomies. They focused on the 10 most productive nations, all high- and middle-income nations. The study highlights that funding for endoscopic spine research is correlated with the publication productivity [13].

### 3.2. Access to Equipment

Limited access to neurosurgical and imaging equipment further restricts FESS implementation in LICs. Karekezi et al. found significant variability in equipment availability among recent neurosurgery graduates in Sub-Saharan Africa (SSA) [11]. Their survey, which included 21 neurosurgeons across 12 LICs in SSA, revealed that only 19.1% had access to endoscopic tools. It should be noted that the survey did not differentiate between spinal or cranial endoscopic tools, making the quantification of access FESS difficult. Additionally, 14.3% of the surveyed neurosurgeons reported having no access to any advanced imaging or surgical equipment. Beyond physical equipment, access to stable internet connections is a prerequisite for remote mentorship, virtual workshops, and case-based teleconsultation.

### 3.3. Research Representation and Productivity

Bibliometric analyses indicate a significant underrepresentation of LICs in FESS-related research. Liu et al. and Wu et al. reported that most publications on FESS originate from high- and middle-income countries, with LICs absent from the list of the most research-productive nations [12,13]. A strong correlation exists between research funding and output, with high-income nations leading in both domains. This lack of FESS research representation in LICs limits opportunities for training, technological advancement, and knowledge dissemination, further hindering the adoption of FESS techniques in resource-limited settings.

The implementation of FESS in LICs is hindered by multiple interrelated barriers, including workforce shortages, lack of infrastructure, lack of access to required equipment, and research underrepresentation. Addressing these challenges requires a coordinated global effort that prioritizes sustainable, context-specific solutions.

## 4. Discussion

### 4.1. Workforce Development and Training Initiatives

One of the foremost barriers to FESS adoption is the shortage of adequately trained neurosurgeons or orthopedic spine-trained surgeons in LICs. Examining the availability of neurosurgeons in LICs provides insight into the workforce density required for advanced surgical techniques. Rahman et al. conducted a survey indicating that although 90% of neurosurgeons trained locally return to LICs, the overall density of specialists remains far below the global target of one neurosurgeon per 100,000 people [9]. Given the already limited number of neurosurgeons, access to those specifically trained in minimally invasive techniques such as FESS is even more constrained, significantly hindering the adoption of these advanced surgical methods.

Tayal et al. aimed to evaluate the efficacy of a neurosurgical workshop in improving neurosurgical endoscopic surgery adoption in a lower-middle-income country [14]. They completed surveys of 24 of the 60 participants of the Neuroendocon 23’ workshop held in Chandigarh, India. They looked at participant preference and comfort levels when performing spinal endoscopic surgery before and after attending the Neuroendocon workshop. The survey looked at participant preferences for endoscopic approach compared to microscopic approach. The authors measured responses to several pathology locations, both in skull base and spine. Of the spine procedures, the participants had a prior preference for microscopic approach compared to endoscopic approach for addressing all lumbar pathologies. The study found that survey participants showed increased confidence in managing spine conditions with an endoscopic approach after participating in the workshop (*p* = 0.04), and a preference for endoscopic technique over minimally invasive technique was statistically significant (*p* < 0.001). There was a unanimous consensus among the surveyed participants that FESS should be promoted in lower-middle-income countries. This article speaks to the interest in the adoption of FESS in resource-constrained settings, while also highlighting the importance of targeted exposure and training in shifting surgeon preferences. Importantly, cultural acceptance of FESS plays a significant role in generating local demand. As demonstrated by survey studies from India, initial hesitancy toward endoscopic approaches—rooted in familiarity with open or microscopic techniques—can be reversed through structured workshops and peer mentorship. These findings emphasize that skepticism toward new technologies may be mitigated by clinical demonstration and locally relevant education. Additionally, healthcare systems in many LICs may favor traditional modalities due to procurement limitations, centralized decision-making, or risk aversion in hierarchical training environments. These contextual factors must be acknowledged in any strategy aimed at increasing FESS adoption. Although this article did not meet the inclusion criteria for low-income countries, it speaks to the aforementioned barriers of FESS adoption in resource-limited countries despite the increasing surgeon interest in FESS adoption.

Pahwa et al. surveyed 160 neurosurgeons in India and compared their preferences for addressing spinal pathologies using either minimally invasive or endoscopic techniques [15]. The study found that neurosurgeons surveyed preferred minimally invasive techniques over endoscopic; the authors attributed this to the steep learning curve associated with FESS. These findings represent potential opportunities for intervention. Addressing this deficit necessitates the expansion of international spine surgery training programs, including fellowship opportunities focused on minimally invasive techniques. Virtual learning platforms, international collaborations, and visiting surgeon programs can supplement local training efforts. With the rise in telemedicine and videoconferencing being an efficient and viable option for international collaborations; we foresee this playing a larger role in helping bridge the learning curve associated with FESS implementation. Strengthening academic partnerships between high-income and low-income countries may facilitate knowledge transfer and provide structured mentorship opportunities.

Regional shortages of spine surgery can also provide insight into the lack of widespread endoscopic adoption. Karekezi et al. surveyed neurosurgeons in sub-Saharan Africa (SSA) and found a density distribution of 0.38 per one million people [11]. Compared to the United States, which has a distribution of 14.7 neurosurgeons per one million people; therefore, there are 39 times fewer neurosurgeons per inhabitant in SSA compared to the United States [9]. The distribution in Southeast Asia is 3.4 per one million people, about four times less than in the United States [10]. Karekezi et al. surveyed recent graduates (*n* = 21) trained by the World Federation of Neurosurgical Societies Rabat Training Center [11]. The article includes statistics from the survey, finding that 90% of the graduates surveyed returned to work in low-income countries, and the remaining 10% in lower-middle income countries in SSA. However, it is important to note that most of the data quantifying the neurosurgeon workforce come from surveys that tend to bias responses from high- and middle-income countries and typically receive fewer responses from LICs, inevitably leading to overgeneralization and a skewed interpretation of the responses. Regardless, these disparities in terms of neurosurgical coverage create a challenging problem. Indeed, it can be implied that in poorly served regions, neurosurgeons function as true generalists, decreasing their bandwidth to adopt “niche” or cutting-edge procedures.

Despite these limitations, several existing programs offer promising avenues for workforce development. The World Federation of Neurosurgical Societies (WFNS) provides international fellowships and short-term observerships with exposure to minimally invasive spine surgery. Organizations such as AO Spine and the Foundation for International Education in Neurological Surgery (FIENS) have led regional and country-specific workshops focused on basic spine surgery skills, some of which have been expanded to include advanced decompression techniques. Additionally, the American Association of Neurological Surgeons (AANS) and the Congress of Neurological Surgeons (CNS) offer global education initiatives and international travel fellowships designed to enhance access to advanced neurosurgical techniques, including minimally invasive spine surgery. These include: The AANS International Visiting Surgeon Fellowship, which allows neurosurgeons from LICs to observe high-volume surgical centers in North America. The CNS Foundation International Observerships support LIC-based surgeons to participate in structured observerships with access to live surgery, cadaver labs, and virtual learning modules.

With the growing global availability of virtual education platforms, there is significant potential to scale these efforts. The success of online learning during the COVID-19 pandemic has demonstrated the feasibility of webinars, livestreamed surgeries, virtual tumor boards, and asynchronous learning modules to cross geographic and financial barriers. These platforms can be tailored to the FESS learning curve through the development of stepwise curricula, endoscopic simulation tutorials, and remote proctoring networks. Programs like AO Spine’s virtual classroom, CNS Nexus, and AANS Online Education can be leveraged or adapted for spine endoscopy training in LICs. Another crucial strategy is the cultivation of regional “training hubs” centers in LICs with basic endoscopic infrastructure and faculty trained through international partnerships. These hubs could serve as incubators for hands-on FESS exposure, fostering local mentorship and context-specific adaptation of techniques. This model has proven successful in global trauma and pediatric neurosurgery initiatives and holds promise in the spine surgery space, particularly when supported by high-income country partners and visiting experts. Equally important is fostering multidisciplinary collaboration between neurosurgeons and orthopedic spine surgeons. In many LICs, spine care is divided between these specialties, but collaboration is essential for optimizing resource utilization and creating unified FESS training tracks. Shared didactic content, cross-disciplinary case conferences, and co-led workshops could streamline training and build collective capacity across surgical disciplines. All together, these strategies, including blending international fellowships, remote mentorship, regional training centers, virtual learning, and cross-specialty training, represent a comprehensive and scalable framework to strengthen the global spine surgery workforce. Such efforts will be essential for ensuring not only the safe and effective implementation of FESS but also the long-term sustainability and autonomy of LIC surgical systems.

### 4.2. Infrastructure and Equipment Access

Limited access to advanced surgical tools and imaging modalities remains a critical bottleneck in the implementation of FESS in LICs. In a survey conducted by Karekezi et al. [11], only 19.1% of facilities in Sub-Saharan Africa had endoscopic tools; and 14.3% of neurosurgeons lacked access to advanced imaging or surgical technology. They reported the availability of different technologies, including access to a computed tomography (CT) scanner (86%), magnetic resonance imaging (MRI) (38%), surgical microscope (33%), and endoscopic tools (19.1%), the lack of access to the equipment necessary for FESS is one of the main barriers to implementation [11]. The authors found that only four of the 21 surveyed surgeons had access to an endoscope. While this is a very limited sample size, it speaks to the general trend of availability of equipment in LICs in SSA. To our knowledge, this is the only available published paper attempting to quantify endoscope availability in LICs, yet no publications in the literature reviewed resulted in the quantification of spine surgeons trained in FESS in LICs.

Investment in cost-effective, portable endoscopic systems may mitigate some of these challenges. A major obstacle includes the high up front cost associated with FESS equipment, these costs are mitigated in the long term through reduction in operation time, fewer complications and reduced hospital stays, the latter being particularly beneficial to low resource settings and it can buffer the strain of recovery from spine surgery on health infrastructure resources. Further work should be performed to reduce the number and price of disposable pieces in endoscopic sets as well, which can add significant cost. Additionally, public–private partnerships should be explored to subsidize the cost of surgical equipment. Exploring local manufacturing of reusable instruments or reprocessing of select disposable tools could reduce long-term operational costs. Mobile surgical units equipped with endoscopic technology could also provide temporary relief in areas with limited healthcare infrastructure. Notably, the advent of biportal endoscopy may at least partially address this issue. Biportal endoscopy allows for a wider range of motion during surgery, dampening the steep learning curve and reducing the cost associated with ultra-specialized equipment and expenditures for specialized training. Several centers around the world have adopted biportal endoscopy precisely for its affordability. While biportal endoscopy does require the use of endoscopic equipment, it can often piggyback on existing arthroscopic equipment used by orthopedic surgeons, thereby decreasing the need for additional capital expenditure.

### 4.3. Research and Policy Advocacy

A critical gap in the literature exists regarding FESS in LICs. Among thousands of articles on FESS, only four addressed LICs. High-income countries dominate publication productivity and funding, leaving LICs with minimal representation. When composing this manuscript, one of the major obstacles was obtaining sufficient data surrounding FESS in LICs. It is well documented that LICs are underrepresented in research as a whole, and this trend is no different when considering research on FESS. A study investigating the publication rates of different nations regarding FESS found that high-income countries (particularly China, Korea, and the United States) were the most productive [12]. Several studies conducted have similarly found confirmatory results illustrating that the majority of publications regarding endoscopic spine surgery have come from middle- and high-income countries in Asia [13]. The lack of representation of low-income countries in the literature is likely emblematic of the limited access to endoscopic spine surgery [13]. Efforts such as international collaboration or targeted funding opportunities in LICs should be considered to help bridge the literature representation gap.

This narrative review highlights the significant challenges low-income countries face in accessing endoscopic spine surgery. Despite its benefits in terms of reduced recovery times, shorter hospital stays, and lower complication rates, the adoption of FESS in LICs is hindered by several barriers, including a lack of trained spine surgeons and insufficient access to equipment. The limited availability of research on FESS in LICs further complicates efforts to improve access, reflecting the broader issue of the underrepresentation of LICs in medical research.

As spinal disorders continue to place a heavy burden on populations in LICs, increasing access to FESS could play a crucial role in improving healthcare outcomes and reducing disability. Future efforts must prioritize bridging the gap in surgical innovation between high-income countries and LICs to ensure equitable healthcare solutions for all. By overcoming these barriers, FESS can become a transformative tool in reducing disparities, enhancing surgical care, and addressing the pressing healthcare needs of underserved populations globally. Encouraging locally conducted research and ensuring that findings are disseminated in high-impact journals will provide valuable data to support policy changes. Funding agencies should prioritize grants for research on FESS in LICs to better understand context-specific challenges and inform evidence-based interventions.

It is important to acknowledge that this manuscript aims to identify barriers to the implementation of endoscopic spinal surgery based on a review of the existing literature. We recognize that this methodology may introduce significant gaps and blind spots in our understanding of the real-world challenges faced in different regions. Furthermore, the low volume of literature representation of FESS in LIC could introduce a bias of overgeneralization for LICs, being that the available literature was particularly in two regions SSA and Southeast Asia. We recognize that this is a limitation of our review. Additionally, language limitations may have excluded relevant publications in non-English languages, potentially underestimating regional efforts. Our intention is not to take a paternalistic stance on this issue; rather, we fully acknowledge that this discussion would be greatly enriched by the perspectives of surgeons who live and practice in these regions.

Additionally, our study may underestimate the true penetrance of FESS in LICs, as orthopedic spine surgeons, who also perform these procedures, are not represented in our data, which are limited to a neurosurgical cohort. Unilateral biportal endoscopy (UBE) has recently gained broader global adoption, in part due to its lower upfront capital investment and reduced disposable costs. Because UBE relies on arthroscopic equipment that is more widely available worldwide, the actual number of spinal surgeons performing endoscopic procedures in LICs may be higher than reported.

We hope this manuscript will serve as a springboard for further collaboration, discussion, and efforts toward broader implementation and adoption of endoscopic spine surgery.

## 5. Conclusions

This narrative review underscores the significant barriers to implementing endoscopic spine surgery in low-income countries, including limited training, inadequate access to essential equipment, and underrepresentation in medical research. Despite these challenges, FESS has immense potential to reduce the burden of spinal disorders in LICs due to its minimally invasive approach, which offers faster recovery, reduced hospital stays, and lower complication rates. Addressing these barriers requires a multifaceted strategy that includes expanding training opportunities, increasing equipment availability, fostering international collaborations, and prioritizing research tailored to LIC contexts. Further research surrounding infrastructure and training availability in LICs is needed to bring forth meaningful advances in FESS implementation in these regions. Support from international collaborations offering virtual trainings and in-person workshops can further advance FESS implementation in LICs. Subsidizing the equipment necessary for FESS can be considered to diminish the upfront cost associated with FESS implementation and allow LIC to benefit from the aforementioned benefits of FESS, reducing healthcare costs in the long term. Coordinated efforts among ministries of health, academic institutions, and industry partners will be essential to sustainably expand access to endoscopic spine care. By bridging the gaps in surgical innovation and resource allocation, FESS can emerge as a critical tool in improving healthcare equity and outcomes for underserved populations, ultimately contributing to the global effort to reduce healthcare disparities.

## Abbreviations

The following abbreviations are used in this manuscript:

FESS	Full endoscopic spine surgery
UBE	Unilateral biportal endoscopy
LIC	Low-income country
LMIC	Lower-middle-income country
UMIC	Upper-middle-income country
HIC	High-income country
SSA	Sub-Saharan Africa

## References

[1] Wu A., March L., Zheng X., Huang J., Wang X., Zhao J., Blyth F.M., Smith E., Buchbinder R., Hoy D. (2020). Global Low Back Pain Prevalence and Years Lived with Disability from 1990 to 2017: Estimates from the Global Burden of Disease Study 2017. Ann. Transl. Med..

[2] World Bank Blogs. https://blogs.worldbank.org/en/opendata/world-bank-country-classifications-by-income-level-for-2024-2025.

[3] Ravindra V.M., Senglaub S.S., Rattani A., Dewan M.C., Härtl R., Bisson E., Park K.B., Shrime M.G. (2018). Degenerative Lumbar Spine Disease: Estimating Global Incidence and Worldwide Volume. Glob. Spine J..

[4] Mahan M.A., Prasse T., Kim R.B., Sivakanthan S., Kelly K.A., Kashlan O.N., Bredow J., Eysel P., Wagner R., Bajaj A. (2023). Full-Endoscopic Spine Surgery Diminishes Surgical Site Infections—A Propensity Score-Matched Analysis. Spine J..

[5] Burkett D., Brooks N. (2024). Advances and Challenges of Endoscopic Spine Surgery. J. Clin. Med..

[6] Chung A.S., Ballatori A., Ortega B., Min E., Formanek B., Liu J., Hsieh P., Hah R., Wang J.C., Buser Z. (2021). Is Less Really More? Economic Evaluation of Minimally Invasive Surgery. Glob. Spine J..

[7] Leyendecker J., Mahan M., Findlay M.C., Prasse T., Köster M., Rumswinkel L., Shenker T., Eysel P., Bredow J., Zaki M.M. (2025). Full-Endoscopic Spinal Decompression or Discectomy Show Benefits Regarding 30-Day Readmission Rates When Compared to Other Spine Surgery Techniques: A Propensity Score Matched Analysis. Spine J..

[8] Lucio J.C., VanConia R.B., DeLuzio K.J., Lehmen J.A., Rodgers J.A., Rodgers W.B. (2012). Economics of Less Invasive Spinal Surgery: An Analysis of Hospital Cost Differences between Open and Minimally Invasive Instrumented Spinal Fusion Procedures during the Perioperative Period. RMHP Risk Manag. Healthc. Policy.

[9] Rahman S., McCarty J.C., Gadkaree S., Semco R.S., Bi W.L., Dhand A., Jarman M.P., Ortega G., Uribe-Leitz T., Bergmark R.W. (2021). Disparities in the Geographic Distribution of Neurosurgeons in the United States: A Geospatial Analysis. World Neurosurg..

[10] Gupta S., Gal Z.T., Athni T.S., Calderon C., Callison W.É., Dada O.E., Lie W., Qian C., Reddy R., Rolle M. (2024). Mapping the Global Neurosurgery Workforce. Part 1: Consultant Neurosurgeon Density. J. Neurosurg..

[11] Karekezi C., El Khamlichi A., El Ouahabi A., El Abbadi N., Ahokpossi S.A., Ahanogbe K.M.H., Berete I., Bouya S.M., Coulibaly O., Dao I. (2020). The Impact of African-Trained Neurosurgeons on Sub-Saharan Africa. Neurosurg. Focus.

[12] Liu Y., Kotheeranurak V., Quillo-Olvera J., Facundo V.I., Sharma S., Suvithayasiri S., Jitpakdee K., Lin G.-X., Mahatthanatrakul A., Jabri H. (2023). A 30-Year Worldwide Research Productivity of Scientific Publication in Full-Endoscopic Decompression Spine Surgery: Quantitative and Qualitative Analysis. Neurospine.

[13] Wu B., Yang L., Fu C., Zhuo Y., Feng X., Xiong H. (2022). Global Trends and Hotspots in Endoscopic Discectomy: A Study Based on Bibliometric Analysis. Neurospine.

[14] Tayal A., Pahwa B., Chaurasia B., Gendle C., Sahoo S.K., Singh A., Gupta S.K., Dhandapani S. (2023). The Call for Neuroendoscopy Cadaveric Workshops in Lower-Middle Income Countries. World Neurosurg..

[15] Pahwa B., Tayal A., Chowdhury D., Umana G.E., Chaurasia B. (2023). Endoscopic versus Microscopic Discectomy for Pathologies of Lumbar Spine: A Nationwide Cross-Sectional Study from a Lower-Middle-Income Country. J. Craniovertebral Junction Spine.

